# The Roles of Different Sources of Social Support on Emotional Well-Being among Chinese Elderly

**DOI:** 10.1371/journal.pone.0090051

**Published:** 2014-03-03

**Authors:** Haifeng Li, Yang Ji, Tianyong Chen

**Affiliations:** 1 Key Laboratory of Mental Health, Institute of Psychology, Chinese Academy of Sciences, Beijing, China; 2 University of Chinese Academy of Sciences, Beijing, China; University of Utah, United States of America

## Abstract

**Background:**

Social support has been widely known as a protective factor for the emotional well-being (EWB) of older adults, but less studies have investigated the roles of different sources of social support (i.e., family and friend support) on different facets of EWB (i.e., positive affect and negative affect) simultaneously.

**Methodology and Findings:**

In this study, the associations between family/friend support and positive/negative affect were investigated in a sample of 700 Chinese elderly. The EWB and social support were measured with a 12-item affective wordlist (Kahneman et al., 2004) and a self-prepared questionnaire. The results showed that (1) the order of contact frequency and mutual support followed a hierarchical order from spouse, children, to friends; (2) zero-order correlations of both family support and friend support were associated with more positive affect and less negative affect; and when compared with the relative role of family and friend support, (3) spouse (children if spouse is not available) support had greater contribution on decreasing negative affect, while friend support had greater influence on increasing positive affect, even after controlling the demographic, self-rated health and life events variables.

**Conclusion:**

Family and friend support play different roles on the two facets of EWB of the elderly. These results were better explained in light of the task specificity model rather than the hierarchical compensatory model. Moreover, positive affect may be enhanced by friend support (based on personal interests and selectable) rather than family support (bonded by kinship and not selectable), which added evidences to the socioemotional selectivity theory.

## Introduction

It is well-known that social support has beneficial effects on subjective well-being of older adults among domains of enjoyment, morale, depression and loneliness [1]–[5]. The sources of social support range from close and stable relations to more distant and unstable relations. As examples, being providers of practical assistance [6]–[8] and emotional needs [9], [10], family members offer core support when older adults were in need [11], [12]; friends, neighbors, and colleagues also play non-negligible roles in terms of socializing and recreation among the elderly people [12]–[14]. Due to the varied roles associated with family and friends, it is worth asking whether various sources of social support of the elderly are related to their subjective well-being in different ways.

Emotional well-being (EWB) refers to the emotional quality of an individual's everyday experience, that is, the frequency and intensity of experiences of joy, fascination, anxiety, sadness, anger, or the affections that would make one's life pleasant or unpleasant. It belongs to one of two aspects of subjective well-being (or hedonic well-being), while the other aspect referring to the general evaluation of life quality [15], [16]. EWB consists of a positive and a negative facet, which were found to be relatively independent of one another [17]–[20]. Yet both positive and negative affect are correlated with subjective well-being constructs, including quality of life and emotional well-being [20], [21]. Unexpectedly, after reviewing the literature, we found very few studies investigated the influences of different sources of social support on positive and negative affect simultaneously. Previous literature on the association between family/friend support and the EWB will be briefly outlined as below.

### Family Support on EWB

Family members are core providers of material aid and instrumental support, who also take on important roles for the EWB of the elderly [12], [13]. On one hand, numerous studies found family support has a strong effect on reducing older adults' negative affect, and this effect is even stronger than it does for friend support. Those elderly women with lower perceived family support were associated with higher negative affect and feeling of loneliness, but their levels of friend support or objective network embedment were not relevant to the negative affect [4]. Similar results were found among Chinese elderly participants, showing satisfaction with family support as the primary predictor for less depressive symptoms compared to objective network relations [22], [23]. It was further discovered that material aid and instrumental support are more important in ameliorating negative affect when provided by family members than provided by friends or acquaintances [24]–[27]. In a longitudinal study among Chinese elderly, those with more depressive symptoms are associated with increased family support but decreased friend support three years after [23]. Such results added weights to the importance of family support on decreasing the negative affect of the elderly.

On the other hand, other studies suggested that family support is helpful in increasing positive affect of the elderly. One American study found that contacting more frequently with family members had a beneficial but weak effect on morale, although relatively less so compared with the effect of interacting with friends [28]. In China, family support similarly was associated with perception of happiness among aged people [29], [30]. Both in western and eastern cultures, nevertheless, the influences of family support on positive affect were less studied.

To better discern the effect of family support on EWB, the characteristic of family support may be of relevance. Family relationship is continuously bonded by aid, affect, and affirmation and is difficult to break apart across lifespan. Empirical data confirmed that intimate, attachment relationships were most frequently occurred within family members [7], [30]–[34], therefore they are often considered as the first choice of the elderly when requesting tangible aid [11], [12].

To sum up, family support is closely associated with the EWB of the elderly [11], [12], [30]–[34]. It is noted that many studies considered family support being of particular relevance to the amelioration of negative affect, and a few studies also showed its relevance to the enhancement of positive affect. In particular, compared with other members of a family, spouse is the core supporter and plays the most important role for providing a buffer for the elderly to reduce negative affect.

### Friend Support on EWB

Apart from family members, friends also play non-negligible roles on the elderly's EWB. While most studies on friend support were concerned with positive affect, relatively fewer studies have focused on negative affect of the elderly.

In terms of positive affect, past studies showed frequencies of contacting with friends were more strongly associated with life satisfaction, happiness, and self esteem of the elderly compared with children support and served as a primary predictor for morale than family support [2]–[4], [28], [35], [36]. In terms of quality of relationships, studies again suggested that older adults reported higher levels of enjoyment and better self-esteem when interacted with friends compared with family members [37]–[39].

When it comes to negative affect, still some studies revealed that support from friends prevents or reduces the feeling of loneliness [36], [40], [41]. Perceived support from both family members and friends were associated with less depressive symptoms [1]. In the context of China, friend support was found unrelated to depression levels, it was suggested that perhaps Chinese older people less relied on friend support for ameliorating negative affect when facing difficulties [22], [42].

Results of friend support on EWB may also be influenced by the characteristic of friend interactions. Compared to family support, friend support is generally considered as great sources for those aspects of EWB related to current state of enjoyment [2]. Networking with friends tends to be a matter of choice, which is spontaneous and reciprocal [35], [36], [43], rather than predetermined as in kinship relations [11], [12], [44]. As birds of a feather flocked together [45]–[47], friends may be more open to leisure activities and conversations of mutual interests, which are oriented towards the enhancement of positive affect [14], [48].

### Related Theories

To clarify whether different sources of social support have the same or different effect on either positive or negative affect, the hierarchical compensatory model and the task specificity model provided two guiding perspectives useful for establishing frameworks in studying effects of social support on different facets of the EWB [49].

According to the hierarchical compensatory model [13], [50], people seek social support from others based on a set of ordered preferences rather than the types of assistance required. Once the initially preferred sources of support are absent or insufficient, individuals from the lower hierarchies would replace them in a compensatory manner [13], [50], [51]. This description bears similarity to the convoy model that the levels of social support reduces as one moved from circles that are closer and more stable (i.e., spouse and children) to ones that are less so (i.e., friends, neighbors, and colleagues) [52]. In contrast, the task specificity model proposed that different groups of people work on specifically tailored tasks or provide different sorts of social support, and if the elderly are deprived of support from certain groups (e.g., friends), their corresponding tasks cannot be substituted or can only be partly supplemented by others.

### The Present Study

Previous literature so far looked at either different source of social support on the same facet of the EWB (e.g., family and friend support on negative affect) or specific type of social support on different facets of EWB (e.g., friend support on positive and negative affect). Despite a few inconsistent results, most studies seemingly suggested that family support outdoes friend support in predicting negative affect levels; and friend support are more important than family support in predicting positive affect levels.

To our knowledge, nevertheless, few except one survey assessed family and friend support simultaneously among 60 older women, and found that family (both spouse and children) support and perceived friend support were predictive of positive affect, and family quality was predictive of negative affect [53]. Yet the survey was limited in sample size and representativeness. Therefore, the question how sources of social support would influence different aspects of the EWB needs to be reassessed through the lens of the hierarchical compensatory model and the task specificity model.

Moreover, from perspectives of cultural norms, family plays a pivotal role in supporting the elderly in China compared with other sources of social support [5], [22], [42]. On the other hand, influenced by filial piety, the preferred living arrangement in China for the elderly remained to be co-residence with children, especially when an older adult's spouse was no longer available [30], [31]. Therefore, children ranked just below spouse in providing critical instrumental support for the elderly [21], [54], [55]. Based on the hierarchical compensatory model, it is of interest to analyze how children support was related to EWB among older adults without spouse than those with spouse in the context of Chinese culture.

The purpose of this study is to compare the relative importance of spouse, children and friend support on positive and negative affect of the EWB. Under the guidance of the theories presented above and based on previous studies, we considered that to be consistent with either the hierarchical compensatory model or the task specificity model, results would likely to be different.

If results were consistent with the hierarchical compensatory model, support from spouse, children, and friends would likely follow a descending order in their associations with both positive and negative affect; and when the spouse was unavailable or absent, it would be replaced by children and friend support in sequence.

Alternatively, if results were consistent with the task specificity model, spouse, children and friend support may be related to positive and negative affect differently; and the effect of spouse, children, and friend support on positive and negative affect may or may not follow a descending order.

## Methods

### Ethics Statement

This research was approved by the Ethics Committee of the Institute of Psychology, Chinese Academy of Sciences. All participants provided written informed consent to participate in the study.

### Participants and Procedures

We finally recruited 700 older adults based on convenient sampling, who came from communities of Haidian (342 persons), Dongcheng (173 persons) and Fengtai (185 persons) districts in Beijing. The voluntary nature of the participation and the confidentiality of their data were assured for the participants. Of those who agreed to participate, another 49 participants were removed from the analyses, since they did not complete the survey. The completion rate was 92.8%. In addition, five respondents were excluded since they were never married, hence cannot be assessed in terms of spouse or children support. We excluded them from the analyses and confirmed that our results were not sensitive to this choice. Interviews were conducted with the participants either at their homes or in the community center. Participants answered the questionnaire by themselves or under the instruction of the investigators. The survey typically took 30 to 45 minutes to complete. Each participant was offered a small token for their participation.

The sociodemographical data of this sample were presented in Table 1. Briefly, the sample was predominantly young old adults, relatively well-educated but financially deprived, which was typical of the urban population in China. Although 13.1% of the participants reported more than two chronic diseases and 13.9% reported in bad or very bad health status, most of the participants were physically healthy and no one was confined to their homes. Most of the participants lived with spouse and/or children, and did not experience any negative life events during the last two weeks. In addition, 21.9% of the participants were widowed/divorced, including 19.9% widowed and 2.0% divorced. Widowed/divorced participants were older, less educated, and poorer in financial status.

**Table 1:** Descriptive Characteristic of Participants with Different Marital Status.

Variables	Married (*n* = 547)	Widowed/divorced (*n* = 153)	All (*n* = 700)
Age (%)			
60–69	63.4	28.8	55.9
70–79	32.2	49.7	36.0
80+	4.4	21.6	8.1
Female (%)	52.3	60.8	54.1
Education level (%)			
0–6 years	15.2	34.0	19.3
7–12 years	64.3	53.2	62.1
13+ years	20.5	11.8	18.6
Income (%)			
< RMB 1000	8.4	18.3	10.6
RMB 1001-3000	75.2	69.3	73.8
≥ RMB 3000	16.5	12.4	15.6
Income satisfaction (%)			
Satisfied or very satisfied	36.7	32.7	35.9
So so	41.3	43.8	41.9
Unsatisfied or very unstisfied	21.9	23.5	22.3
Number of chronic disease			
none	26.5	19.6	25.0
1	40.4	43.1	41.0
2	20.3	22.9	20.9
3+	12.8	14.4	13.1
Self-rated health status (%)			
Good or very good	24.3	16.3	22.6
So so	64.0	62.1	63.6
Bad or very bad	11.7	21.6	13.9
Living arrangement (%)			
With spouse and children	40.8		31.9
With spouse only	49.2		38.4
With children only	7.7	62.1	19.6
Alone	2.4	37.9	10.1
Life events (%)			
None	84.5	75.2	82.4
At least one	15.5	24.8	17.6

### Measurement

**Family and Friend Support:** Social support was measured by three questions as below, namely (1) how frequently have you contact with him/her/them? (2) how extensive have he/she/they provide support to you? (3) how extensive have you provide support to him/her/them? Participants were asked to rate the contact frequency and mutual support under (a) spouse, (b) children, (c) friends/neighbors/colleagues. Questions were rated on a 5-point Likert scale, ranging from 1 (very rarely) to 5 (very frequently). Participants without spouse or children rated as 0 (not available) in questions regarding to spouse or children support respectively. In this study, social support was represented as the average score of these three questions.

For those elderly with spouse, family support included spouse support and children support, while for widowed or divorced elderly, family support is represented only by children support. In China, the concept of “friends” is defined as “those who shared a good relationship with each other” according to the Contemporary Chinese Dictionary (the 6th edition, 2013). In fact, most friends “who shared a good relationship with each other” are the colleagues and neighbors of the Chinese elderly. Thus the ambiguous definition of friends was extended to “friends/colleagues/neighbors who shared a good relationship with you” as a specified working definition in the present study. On the other hand, the roles of friends, neighbors, and colleagues to the elderly may be overlapped. It was regarded friend support and neighbor support as a similar support form [56]. For most people, both friends and colleagues could provide companionship and emotional support to the elderly [57], [58]. In the later section of this article, “friends/colleagues/neighbors who shared a good relationship with you” were named after “friends” for short.

The internal consistency of the social support items was adequate in its entire form (Cronbach *α* = .84) or in terms of spouse, children, and friend support (Cronbach *α* between 0.83 and 0.98). The test-retest reliability in 44 participants during one month interval was 0.94 (*p*<0.001).

**EWB: The Positive and Negative Affect:** The EWB was measured by a 12-item affective wordlist, which was taken from Kahneman et al. [59]. Four word items are related to positive affect and eight word items are related to negative affect. Participants were asked to rate the 12 words or phrases they experienced during the past month, for example, happy, depressed/blue, impatient and so on. The scale rated on a 5-point Likert scale, ranging from 0 (never) to 4 (always). Positive affect is the average of ratings on *happy*, *warm*/*friendly*, *enjoyment myself*, *competent*. Higher score represents a more positive affect. Negative affect is the average of ratings on *frustrated/annoyed*, *depressed/blue*, *hassled/pushed around*, *angry/hostile*, *worried/anxious*, *criticized/put down*, *impatient*, *tired*. Higher score represents a more negative affect. In this study, the internal consistency (Cronbach α) of positive affect and negative affect items were 0.75 and 0.87 respectively.

**Sociodemographic Information and Other Variables:** The sociodemographic information (age, sex, marital status, education level, monthly income and income satisfaction), health status (number of chronic diseases, self-rated health status), living arrangement, and life events were included in the survey. Income satisfaction was measured on 5-point scale ranging from very unsatisfactory to very satisfactory. The list of chronic diseases included hypertension, coronary disease, cerebrovascular disease, diabetes, chronic bronchitis, spondylosis or osteoarthrosis, pulmonary disease (asthma, emphysema or cor pulmonale), cancer, and others (if not in the list). Self-rated health status was measured on 5-point scale ranging from very good to very bad. The list of life events included serious disease or injury of oneself, die of relatives, serious disease or injury of family members, children divorce, die of friends, bad relationship with spouse, bad relationship with children, suffering loss by theft or property loss, unforeseen events (unexpected frightening, having an accident, or suffering from natural disaster). In order to avoid collinearity problems, only age, sex, education, self-rated health, and life events were included in the final regression model.

## Results

The structure of the social support questionnaire was illustrated by an exploratory factor analysis. Based on both the eigenvalue and the turning point of the scree plot, two factors were identified among the widowed/divorced, and three factors were identified among the married, which in turn explained 76% and 77% of the total variation. Furthermore, these factors corresponded to the social support from spouse, children, or friends as shown in Table 2.

**Table 2:** The Factor Loadings of the Social Support Questionnaire for Different Marital Status.

Question	Widowed/divorced (n = 153)		Married (n = 547)		
	Factor 1	Factor 2	Factor 1	Factor 2	Factor 3
Spouse-CF			**0.817**	0.025	0.325
Spouse-RS			**0.869**	0.052	0.242
Spouse-PS			**0.862**	0.156	0.224
Children-CF	0.200	**0.860**	0.046	**0.786**	0.207
Children-RS	0.178	**0.879**	0.082	**0.863**	0.158
Children-PS	0.397	**0.716**	0.096	**0.862**	0.197
Friends-CF	**0.801**	0.261	0.304	0.186	**0.832**
Friends-RS	**0.834**	0.230	0.248	0.257	**0.817**
Friends-PS	**0.880**	0.209	0.372	0.281	**0.705**

Note. CF  =  contact frequency; RS  =  received support; PS  =  provided support.

For the levels of social support, as illustrated in Table 3, spouse, children and friends hierarchically followed a descending order in terms of contact frequency, provided support and received support among married elderly; similarly when spouse was no longer available, support from children was greater than those from friends.

**Table 3:** Contact Frequency and Social Support Exchange for Different Marital Status.

	Married (n = 547)	Widowed/divorced (n = 153)
Contact frequency		
Spouse	4.32 (0.98)	
Children	3.99 (0.93)	3.87 (0.89)
Friends	3.37 (0.93)	3.26 (0.99)
Provided support		
Spouse	4.32 (0.93)	
Children	3.94 (0.90)	3.84 (0.92)
Friends	3.13 (0.95)	3.14 (1.09)
Received support		
Spouse	4.23 (0.95)	
Children	3.95 (0.92)	3.83 (0.98)
Friends	3.12 (0.93)	3.06 (1.03)

Note. Standard deviations appear in parentheses.

To illustrate the associations between social support and the EWB, zero order correlations between levels of different social support and the subjective ratings of both positive and negative affect, among married and widowed/divorced older adults, were presented in Table4. From spouse, children, to friend support, the sizes of correlation coefficients followed a descending order for positive affect and an ascending order for negative affect. Again, this trend was replicated among widowed/divorced older adults. All correlation results reached statistical significance.

**Table 4:** Correlations between Social Support and Emotional Well-being.

	Married (n = 547)		Widowed/divorced (n = 153)	
	PA	NA	PA	NA
Spouse support	.18***	−.35***	—	—
Children support	.30***	−.27***	.20*	−.34***
Friend support	.43***	−.13**	.41***	−.21**

Note. PA  =  Positive affect; NA  =  Negative affect.

*p<0.05. **p<0.01. ***p<0.001.

Two multiple regression models were performed to examine the effect of social support on different facets of the EWB (Table 5). After attributing levels of spouse, children, and friend support in Model 1, results suggested that levels of spouse support was the only significant predictor for negative affect ratings, and the levels of friend support was the only significant predictor for positive affect ratings among married older adults; similarly, children support replace spouse support to become the only significant predictor on negative affect ratings among widowed/divorced older adults. In Model 2, demographical factors as age, gender, and education levels, along with self-rated health status, and life event factors were added, findings for the effects of social support on EWB were found not to differ from Model 1 results.

**Table 5:** Multiple Regressions of Positive and Negative Affect on Social Support, Demographic Information, Health and Life Events.

		Positive Affect			Negative Affect		
		*Beta*	*R^2^*	*F*	*Beta*	*R^2^*	*F*
Married (n = 547)							
Model1	Spouse support	.04	.19	43.24***	−.30***	.13	26.27***
	Children support	.09			−.07		
	Friend support	.37***			−.03		
Model2	Spouse support	.02	.26	16.86***	−.26***	.19	11.64***
	Children support	.08			−.07		
	Friend support	.32***			.01		
	Age^a^						
	70–79	.01			−.05		
	80+	−.13***			−.03		
	Male	−.03			−.04		
	Education^b^						
	7–12 years	.05			.07		
	13+ years	.05			.06		
	Self-rated health^c^						
	So so	−.16***			.12**		
	Bad or very bad	−.24***			.15**		
	Life events	.02			.20***		
Widowed/divorced (n = 153)							
Model1	Children support	−.04	.17	15.34***	−.32***	.12	9.97***
	Friend support	.43***			−.04		
Model2	Children support	.00	.29	5.91***	−.29**	.23	4.29***
	Friend support	.36***			−.05		
	Age^a^						
	70–79	−.17*			−.10		
	80+	−.21*			−.10		
	Male	.03			.06		
	Education^b^						
	7–12 years	.09			.11		
	13+ years	.14			.12		
	Self-rated health^c^						
	So so	−.11			.15		
	Bad or very bad	−.18			.29**		
	Life events	−.18*			.24**		

Note. ^a^ The reference to age was 60–69 years; ^b^ the reference to education was 0–6 years; ^c^ the reference to self-rate health was good or very good.

*p<0.05. **p<0.01. ***p<0.001.

Additional to Model 1 results, Model 2 found age, health, and life event factors were more or less associated with positive and/or negative affect levels, which was consistent with previous literature [60], [61]. In terms of age, results showed that being older (80+ years of age) was associated with less positive affect among married older adults. This trend was similarly found among widowed/divorced older adults if they were older (70–79 and 80+ years of age). In terms of self-related health, less positive affect and more negative affect were associated with worse-off health conditions (“so so”, and/or “bad or very bad”) among married older adults. Results among widowed/divorced older adults were similar in direction without reaching statistical significance, perhaps due to the small within group sample sizes. Last but not least, negative life events were associated with lower positive affect ratings for widowed/divorced older adults, and higher negative affect ratings for both married and widowed/divorced older adults at significant levels.

Lastly, multiple regressions of positive and negative affect on each aspect of social support, namely contact frequency, received support and provided support, were individually analyzed and the results were consistent when the combined scores were used for the married and the widowed/divorced elderly with only one exception. That is, the association with negative affect was only significant in friends compared to children in widowed/divorced elderly. Since majority of adult children co-reside with their elderly parent/s in China [55], in such situations, mutual support rather than contact frequency might be a better index of the actual social support from children. Indeed, the associations of mutual support (received and provided support) and negative affect favored children rather than friends. In another attempt, we entered mutual support from children as well as contact frequency from friends into one regression model; again the results favored children (*Beta*  = −.28, *p*<.001) rather than friends (*Beta*  = −.16, *p* = .059).

## Discussion

The purpose of this study was to see how different sources of support were related to EWB. We simultaneously investigate the associations of spouse, children, and friend support on both positive and negative affect, which has been less attempted in previous literature.

Our results firstly demonstrated that levels of social support followed a hierarchy descending order from spouse, children, to friends, among measures of contact frequency, provided and received support. When spouse was unavailable, children still provided more support than friends. These results were consistent with the convoy model [52]. In other words, closer and more stable circles (i.e., spouse followed by children) compared with less close and stable circles (i.e., friends/neighbors/colleagues) were more frequently contacted and mutually supported.

Results showed that family support played an important role in buffering negative affect, and spouse support was replaced by children support when the elderly ceased to be in a married relationship. It further signified the critical contributions of family support in ameliorating the negative aspects of the EWB over and above friend support. Results were consistent with past studies on the association between family support and negative affect [23], [25]–[27], [42], even after controlling for friend support [22].

The reasons for the relatively more important role of family support on negative affect can be tackled by the hierarchical compensatory model [13], [50]. Bonded by intimacy and kinship norms, family tend to be closer and more stable compared with friends [52]. Higher levels of family support may be contemplated as associated with the reception of more instrumental [12], [13] and emotional support [22], [23], [25]–[27], which served as a buffer for negative life events and affective experiences; low levels of family support (i.e., less frequent contact and interactions) provided less buffer for negative life events, and could further imply incidents of family conflicts, it may be due to such reasons that these older adults subsequently reported higher levels of negative affect.

Inconsistent with the findings of western studies [1], [42], our studies found that the friend support did not play a similarly important role in terms of negative affect compared to family support. From a cultural perspective, family was regarded as being pivotal to the support of Chinese elderly, as what might be considered in other cultures [22], [42], [62]. It is generally believed that family has the obligation to look after the elderly when needed; while negative experiences were often not brought up with friends as one would not want to add burdens to others [22], [42]. This view was further supported from the aforementioned longitudinal study that Chinese elderly with more depressive symptoms later received more support from family members yet less from friends [23]. On the other hand, according to filial piety, children also played more important roles to support the older adults compared with friends in China as compared with America [42]. Particularly, when support from spouse was inaccessible, children are often required to provide replacement roles by co-residing with the elderly [31], [54], [55].

Quite unexpectedly, although contact frequencies or mutual support from family members were all higher than those from friends, friends played a more important role in positive affect, inconsistent with the hierarchical compensatory mode [13], [50]. The results found that those with more support from friends reported higher levels of positive affect, which replicated the previous literature [2], [3], [28], [35], [36]. According to the characteristics of friend interaction, friends are oriented towards leisure activities and conversations of mutual interests for increasing positive affect [44]–[48], and together with discussions on family support in the above paragraph [22], [30], [42], friends and family members are assigned different roles to the older adults. Taken together, the differential associations of social support and two aspects of the EWB were perhaps attributed by the specific roles of social members proposed by the task specificity model [11].

Besides, rather than determined by kinship bonds and norms [44], the makings of friends are normally a matter of personal choice [35], [36], [43]. The socioemotional selectivity theory (SST) [63], [64] further suggested that even though older adults gradually abandoned some of their social roles [63], [64], they can actively select, create and manage their social networks by interacting with individuals similar in interests to achieve emotional satisfaction. Thus, the relative more important roles of friends in terms of positive affect perhaps can be explained accordingly by the SST.

The SST was supported from several empirical studies with regards to friend support. For instance, older adults tend to find friends who are similar in interests [45]–[47], and their activities with friends were oriented towards to enhancement of positive affect [14], [48]. Friend support being the primary predictor for positive affect thus supported the agentic roles of the elderly in realization of emotional satisfaction [63], [64]. In our study, higher levels of friend support may be indicative to successful selection and maintenance of social networks for experiencing positive affect, while lower levels could be portraying less successful attempts. Moreover, such effect was not only found among older adults who lived without spouse, but also those who did, which confirmed that friends play more important roles than family members on positive affect.

Lastly, although family support in general was associated with positive affect (nevertheless weaker than those of friend support), it did not show specific contributions to positive affect when friend support was also considered. The results were congruent with the above discussions that sources of social support differentially influenced on positive affect in light of the task specificity model [11] and the SST [63], [64]. Moreover, it is noted that family support are especially considered as voluntary or expected in the sociocultural context of China [62], thus more family support (both spouse and children) may not be necessarily perceived by the older adults as relevant to the enhancements of their positive affect.

### Limitations

Self-report rather than objective measures of social support were used in the current study, thus the perceived social support might not reflect the actual situation. Under the cultural background which emphases family solidarity and filial piety, participants might have elevated the ratings for spouse and children support compared with friend support. When such inflations were removed, the magnitude of the hierarchy might be lessened or even becoming negligible. In addition, we admit that our loose definition of friends was distinctively differed to “close friends” or “best friends” as so defined in some previous studies, thus results cannot be generalized to such alternative definitions. Furthermore, the sample was recruited using convenient sampling from a group of urban dwelling older adults. A related issue was that most of participants were physically healthy, thus the generalizability of our study to China and other societies remains to be investigated. Finally, although the task specificity model was supported from our cross-sectional data, longitudinal data might provide fairer comparisons between the task specificity model and the hierarchical compensatory model.

### Implications

Our study discovered that children could substitute the supportive roles for relieving negative affect when the spouses were not available; while friends play more important roles for enhancing positive affect. These results suggested that, in the context of China, advocating filial responsibilities remained to be a feasible option to ease the burden of social endowment; and furthermore, public programs should take considerations of establishing and strengthening community infrastructure and function to make communication more convenient among the elderly community dwellers.

## Conclusions

Although the results showed that spouse, children, and friend support followed a hierarchical order in consistent with the Convoy model, family and friend support play different roles on the two facets of EWB of the elderly. These results were better explained in light of the task specificity model rather than the hierarchical compensatory model. Moreover, our study showed positive affect may be enhanced by friend support (based on personal interests and selectable) rather than family support (bonded by kinship and not selectable), which added evidences to the SST.
